# Quercetin Prevents Intestinal Stem Cell Aging via Scavenging ROS and Inhibiting Insulin Signaling in *Drosophila*

**DOI:** 10.3390/antiox12010059

**Published:** 2022-12-27

**Authors:** La Yan, Xiaoxin Guo, Juanyu Zhou, Yuedan Zhu, Zehong Zhang, Haiyang Chen

**Affiliations:** 1 Department of Oncology, National Clinical Research Center for Geriatrics, West China Hospital, Sichuan University, Chengdu 610000, China; 2 Laboratory of Metabolism and Aging Research, National Clinical Research Center for Geriatrics, West China Hospital, Sichuan University, Chengdu 610000, China

**Keywords:** intestinal stem cell, aging, quercetin, insulin signaling, ROS, *Drosophila*

## Abstract

Adult stem cells, a class of cells that possess self-renewal and differentiation capabilities, modulate tissue regeneration, repair, and homeostasis maintenance. These cells undergo functional degeneration during aging, resulting in decreased tissue regeneration ability and increased disease incidence. Thus, it is essential to provide effective therapeutic solutions to preventing the aging-related functional decline of stem cells. Quercetin (Que) is a popular natural polyphenolic flavonoid found in various plant species. It exhibits many beneficial effects against aging and aging-related diseases; however, its efficacy against adult stem cell aging remains largely unclear. *Drosophila* possesses a mammalian-like intestinal system with a well-studied intestinal stem cell (ISC) lineage, making it an attractive model for adult stem cell research. Here, we show that Que supplementation could effectively prevent the hyperproliferation of ISCs, maintain intestinal homeostasis, and prolong the lifespan in aged *Drosophila*. In addition, we found that Que could accelerate recovery of the damaged gut and improve the tolerance of *Drosophila* to stressful stimuli. Furthermore, results demonstrated that Que prevents the age-associated functional decline of ISCs via scavenging reactive oxygen species (ROS) and inhibiting the insulin signaling pathway. Overall, our findings suggest that Que plays a significant role in delaying adult stem cell aging.

## 1. Introduction

Most tissues can regenerate and repair in a physiological or damaged state, a process achieved by resident stem cells [1,2]. Adult stem cells can self-renew and differentiate into various cell types to maintain tissue homeostasis [3,4,5]. The loss of tissue homeostasis is caused, at least partially, by a decline in the stem cell number or function, commonly referred to as stem cell exhaustion [6,7,8]. Impaired stem cell function, a well-recognized hallmark of tissue and organismal aging, has been implicated in the development of many aging-related diseases [9,10]. Over the years, numerous studies have explored how stem cells maintain organismal homeostasis and the mechanisms involved in the deterioration of stem cells during aging [11,12]. Scientists have also focused on discovering effective treatment strategies to improve the function of aged stem cells and promote healthy aging. In recent years, studies have revealed that some natural compounds can prevent an aging-associated decline in stem cell function and maintain tissue homeostasis [13,14]. However, the number of these studies is still limited, and the mechanisms through which the compounds act on stem cells have not yet been fully elucidated.

Quercetin (3,3′,4′,5,7-pentahydroxyflavone) is a well-studied polyphenolic flavonoid compound that presents in various vegetables and fruits [15]. As a major flavonoid, Que exhibits abundant biological activities, including antioxidant, anti-inflammatory, anti-cancer, anti-viral, anti-microbial, and anti-protozoal [16]. Consequently, it is effective against multiple diseases, such as cancers, cardiovascular diseases, diabetes, and neurological diseases [17]. Recently, Que has been recognized as a geroprotective compound that eliminates senescent cells and alleviates aging-related disorders [18,19,20]. In addition, studies have shown that Que supplementation can extend the lifespan of mice and *Caenorhabditis elegans* [21,22,23]. The compound has also been reported to stimulate the proliferation of human mesenchymal stem cells (hMSCs) and prevent premature aging [18]. Therefore, the main aim of this study was to explore whether Que could regulate the function of adult stem cells to exert its anti-aging effects and elucidate its mechanism of action.

**Figure 1:**
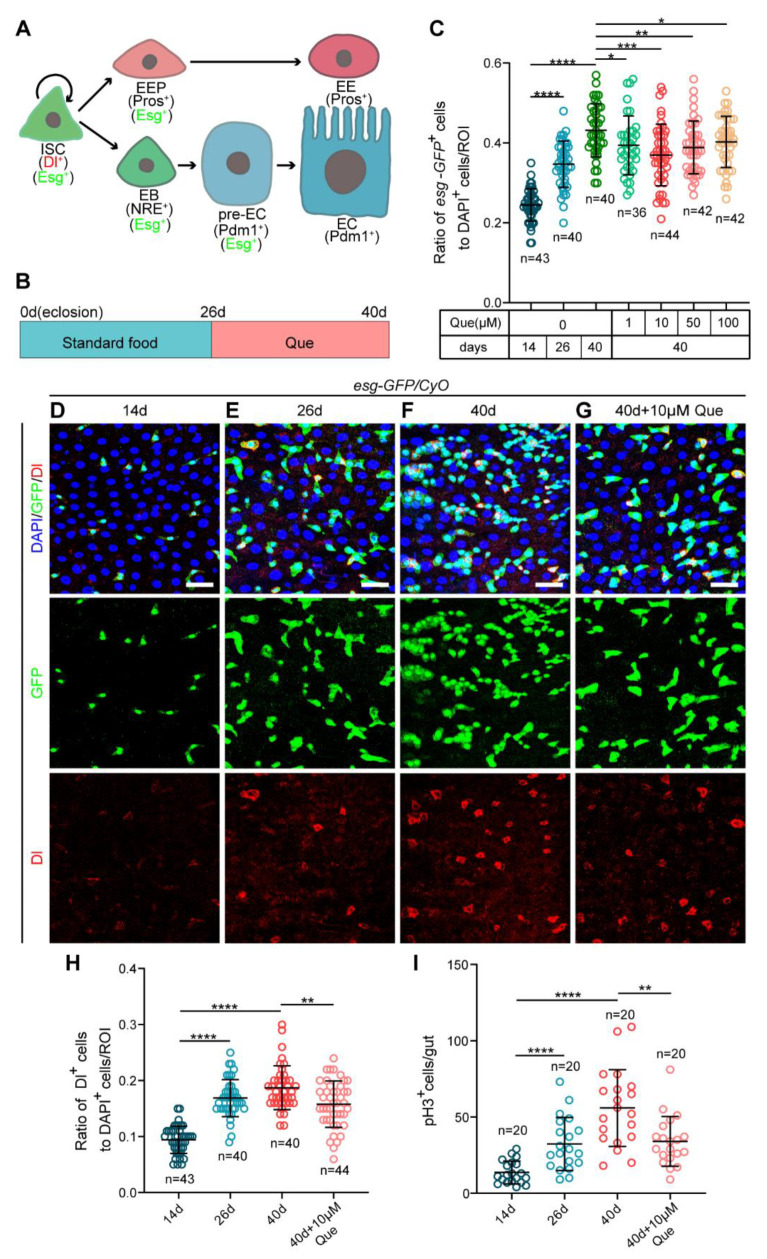
Que prevents gut hyperplasia in aged *Drosophila*. (**A**) The *Drosophila* intestinal stem cell (ISC) lineages. ISCs (marked by Dl and Esg) divide asymmetrically to renew themselves and give rise to enteroendocrine progenitor cells (EEPs; marked by Esg and Pros) or enteroblasts (EBs; marked by Esg and NRE). EEPs further differentiate into enteroendocrine cells (EEs; marked by Pros), whereas EBs with high Notch signaling (pre-ECs; marked by Esg and Pdm1) further differentiate into enterocytes (ECs; marked by Pdm1). (**B**) Schematic diagram showing the process of feeding quercetin (Que) to *Drosophila*. (**C**) The ratio of *esg-GFP*^+^ cells to DAPI^+^ cells per region of interest (ROI) in the posterior midguts of 14-, 26-, and 40-day-old flies (*esg-GFP/CyO*) without Que supplementation and 40-day-old flies fed with four concentrations (1, 10, 50, and 100 µM) of Que. n: number of ROI counted. (**D**–**G**) Representative immunofluorescence images of 14- (**D**), 26- (**E**), and 40-day-old (**F**) posterior midguts without Que supplementation and 40-day-old posterior midguts with 10 µM Que supplementation (**G**) stained with DAPI (blue; nuclei), GFP (green; ISCs and progenitor cells marker), and Dl (red; ISCs marker). The top panels represent the merged images, the middle panels represent *esg-GFP*, and the bottom panels represent Dl. Scale bars represent 25 µm. (**H**) The ratio of Dl^+^ cells to DAPI^+^ cells per ROI in the posterior midguts of flies in experiments D-G. n: number of ROI counted. (**I**) The number of pH3^+^ cells in the whole guts of flies in experiments D-G. n: number of guts counted. Error bars represent standard deviation (SDs). Student’s *t*-tests, * *p* < 0.05, ** *p* < 0.01, *** *p* < 0.001, and **** *p* < 0.0001.

*Drosophila melanogaster* is a well-established model system for studying developmental biology and aging because it possesses the advantages of abundant genetic resources, simple operation, and high similarity with mammalian tissues [24,25]. Over the past decades, the discovery of the intestinal stem cell lineage in the *Drosophila* gut has made it an excellent model for adult stem cell research [4]. The *Drosophila* midgut epithelium comprises several types of cells: intestinal stem cells (ISCs) identified by expressing Delta (Dl, a ligand for the Notch receptor) and Escargot (Esg, a SNAIL family transcription factor); progenitor cells, including enteroblasts (EBs) and enteroendocrine progenitor cells (EEPs); and differentiated cells including enterocytes (ECs) and enteroendocrine cells (EEs). Notably, ISCs are mostly quiescent during intestinal homeostasis, but when the *Drosophila* midgut is stimulated, they divide to self-renew and generate EBs or EEPs. Subsequently, EBs with high Notch signaling differentiate into ECs, whereas EEPs differentiate into EEs (Figure 1A) [4,5,26,27]. Accumulating evidence has revealed that signaling pathways, such as insulin [28,29,30], ROS [31,32], and JNK [33], control the division and differentiation of ISCs and maintain intestinal homeostasis. Studies have also shown that the uncontrolled proliferation of ISCs in the aged *Drosophila* intestine leads to the accumulation of ISCs and progenitor cells (EBs and EEPs), ultimately disrupting intestinal homeostasis and impairing intestinal function [32,33]. Interestingly, the decreased proliferative activity of ISCs in the aged *Drosophila* intestine correlates with extended lifespan and maintenance of intestinal homeostasis [28,34]. Therefore, considering the above, we chose the *Drosophila* midgut as the preferred model tissue for studying the effect and mechanism of Que on ISC aging.

This study found that Que supplementation could reduce the hyperproliferation of ISCs, maintain intestinal homeostasis, and prolong the lifespan of aged *Drosophila*. Furthermore, we demonstrated that Que exerts these effects by inhibiting the insulin signaling pathway and scavenging reactive oxygen species (ROS). Overall, this study provides a new perspective for understanding stem cell aging, which will aid in developing anti-aging strategies.

## 2. Materials and Methods

### 2.1. Drosophila Culture and Strains

All flies were maintained on standard cornmeal-agar food (80 g sucrose, 50 g cornmeal, 20 g glucose, 18.75 g yeast, 5 g agar, and 30 mL propionic acid dissolved in 1 L of water) and kept at standard conditions (25 °C, 60% relative humidity, and 12 h light/dark cycle) unless otherwise mentioned. All flies used in this study were mated females.

The following *Drosophila* lines were used: *esg-GFP/CyO* line, *UAS-lacZ* line and *tub*-*Gal4 line* (from Allan Spradling), *esg^ts^-Gal4* line (from Benjamin Ohlstein), *w^1118^* line (BDSC# 3605), *UAS-Cat* line (BDSC# 24621), *Keap1 RNAi* line (VDRC# v330323), *UAS-InR-DN* line (BDSC# 8253), *InR RNAi* line (BDSC# 51518). The *Drosophila* genotypes used in this study are listed in Table S1. 

The *UAS/Gal4* system was used in this study. Notably, the temperature-sensitive *Gal4* (*esg^ts^-Gal4*)-mediated knockdown or overexpression of genes was repressed at 18 °C and activated at 29 °C. 

### 2.2. Immunofluorescence and Microscopy

The guts were dissected in cold 1 × phosphate-buffered saline (PBS), fixed at room temperature for 30 min with 4% paraformaldehyde (PFA), and then washed three times in 0.1% PBST (PBS plus Triton X-100, BioFroxx, Einhausen, Germany) for 10 min each. Next, the tissues were blocked with 0.5% BSA for 30 min and incubated overnight, with the primary antibodies diluted in 0.1% PBST at 4 °C. On the next day, the guts were washed three times in 0.1% PBST for 10 min each and incubated with secondary antibodies and DAPI for 2 h at room temperature. Finally, the guts were washed three times in 0.1% PBST for 10 min each.

Table S2 shows the primary antibodies and dilutions used in this study. The secondary antibodies and dilutions were as follows: Goat anti-chicken Alexa 488 (Invitrogen, Shanghai, China, 1:2000), Goat anti-mouse Alexa 568 (Invitrogen, 1:2000), and Goat anti-rabbit Alexa 568 (Invitrogen, 1:2000) antibodies. The nuclei were stained using 1 µg/ml 4,6-diamidino-2-phenylindole (DAPI; Sigma, Shanghai, China).

Immunofluorescence images were captured using a Leica TCS-SP8 confocal microscope and then processed using Leica Application Suite X (LAS X, Weztlar, Germany), Adobe Illustrator, and ImageJ software. 

### 2.3. Quercetin, Paraquat (PQ), and Bleomycin (BLM) Treatments

Quercetin (Aladdin, Shanghai, China, #Q111273) was first dissolved in DMSO and then added to standard food and mixed thoroughly to obtain different concentrations (1, 10, 50, 100 µM). Flies were collected randomly and distributed equally into vials containing Que or DMSO (as control) mixed food. 

For the paraquat (Aladdin, #M106761) and bleomycin (Aladdin, #B107423) treatments, a chromatography paper was cut into 3.7 × 5.8 cm strips and soaked in 500 µL of 10 mM PQ or 25 µg/mL BLM. Flies were starved for 1 h before treatment, then transferred into vials with PQ or BLM-soaked chromatography paper strips.

### 2.4. Lifespan Analysis

To determine the effect of Que on the lifespan, 100 female flies hatched within 48 h were collected and divided equally into five vials, in which the food was mixed with DMSO (as control) or Que. In addition, five males were added to each vial to ensure the normal mating of females. The number of dead flies was recorded every one or two days, and at least three independent experiments were analyzed for each group. 

To determine the lifespan under stressful conditions, the flies were randomized into the following groups: PQ (20 mM) + DMSO, PQ (20 mM) + Que (10 µM), BLM (5 µg/ml) + DMSO, and BLM (5 µg/ml) + Que (10 µM) group. For each group, 100 female flies hatched within 48 h were collected and divided equally into five vials. The number of dead flies was counted every one or two days, and at least three independent experiments were analyzed for each group.

### 2.5. Bromophenol Blue Assay

The bromophenol blue assay was performed to determine the pH of *Drosophila* midguts following a previously described protocol [35]. Briefly, 200 µL of 2% bromophenol blue sodium (Sigma, #B5525) was added to the food surface, followed by punching several holes with a pipet tip to allow complete absorption of the solution. Flies pretreated with or without Que were starved for 2 h and then cultured in the prepared food vial for one day. Finally, the guts were dissected and images were captured immediately.

When flies ingested food containing bromophenol blue, their guts were dyed blue, which we call “Eating” flies. A “Non-eating” fly is one that did not eat food and whose gut was not stained with bromophenol blue. We counted the percentage of guts that were stained blue as “Eating (%)”.

### 2.6. Fly Excretion Measurement

The fly excretion assay was performed to test the intestinal defecation behavior, as previously described [36]. Briefly, flies pretreated with or without Que were starved for 2 h and then cultured in the bromophenol blue food vial (whose wall was surrounded by chromatography paper) for one day. Finally, the deposits (each blue dot is a deposit) on the paper were imaged with a Leica M205 FA stereomicroscope and the number of deposits was quantified.

### 2.7. Smurf Assay

The smurf assay was performed to evaluate the intestinal integrity as previously described [37]. First, blue food dye (Spectrum Chemical Manufacturing Corp, Shanghai, China, #FD110) was added to the standard food at a concentration of 2.5% (*wt*/*vol*). Next, flies pretreated with or without Que were cultured in the prepared medium for 12 h. Flies showing blue dye outside the digestive tract were considered “Smurf (+)” flies. 

### 2.8. FACS and RT-qPCR

Midguts were dissected in cold DEPC (diethyl pyrocarbonate)-PBS and incubated with Trypsin (Gibco, Shanghai, China, #15400054) for 1 h at room temperature with gentle shaking. Samples were centrifuged at 2000 rpm for 20 min at 4 °C and filtered using 70 µm filters. Next, *esg-GFP*^+^ cells were collected by flow cytometry (FACS Aria Ⅲ, BD Biosciences, Franklin Lakes, NJ, USA). Each sample contained at least one hundred female midguts.

Total RNA was extracted using an RNA-easy Isolation Reagent (Vazyme, Nanjing, China, #R701-01), followed by the reverse transcription of 1 μg RNA into cDNA using an *Evo* M-MLV RT Kit (Accurate Biology, #AG11711) according to the manufacturer’s instructions. RT-qPCR was performed using ChamQ Universal SYBR qPCR Master Mix (Vazyme, #Q711-02) with a CFX96^TM^ Real-time PCR System (BIO-RAD). Relative mRNA expression was normalized to Rp49 mRNA and calculated using the 2^−ΔΔCt^ method. The primers used in this study are listed in Table S3.

### 2.9. Dihydroethidium (DHE) Staining

DHE staining was performed to determine the levels of ROS in live tissue, as previously described [32]. Briefly, the guts were dissected in Schneider’s medium and incubated in 30 µM DHE (MKbio, Shanghai, China, #MX4812) for 10 min. The guts were then washed three times in Schneider’s medium at room temperature. Images were captured with a Leica TCS-SP8 confocal microscope immediately after DHE staining, followed by calculating the fluorescence intensity using the ImageJ software.

### 2.10. TUNEL Assay

The *Drosophila* midguts were dissected in PBS and fixed with 4% paraformaldehyde for 1/2 h, followed by washing with 0.2% PBST (PBS plus Triton X-100) for 10 min each. Apoptosis was assessed using the TUNEL BrightRed Apoptosis Detection Kit (Vazyme, #A113-01) according to the manufacturer’s instructions.

### 2.11. RNA-Seq

The midguts of adult flies were first dissected in cold DEPC-PBS. Then, the total RNA of midguts was extracted using TRIzol Reagent (Invitrogen, #15596026). Each sample contained 200 female midguts. A total of 2 µg of total RNA was used for the construction of sequencing libraries. PCR products corresponding to 200–500 bps were enriched, quantified, and finally sequenced on DNBSEQ-T7 sequencer (MGI Tech Co., Ltd. Shenzhen, China) with the PE150 model. 

Raw sequencing data were first filtered by Trimmomatic (version 0.36), the de-duplicated consensus sequences were mapped to *Drosophila Melanogaster* ISO1 using the STAR software (version 2.5.3a), read count extraction and normalization were performed using featureCounts (Version 1.5.1), and then RPKMs were calculated. A *p*-value < 0.05 and fold-change > 2 were used to judge the statistical significance of gene expression differences. Gene Ontology (GO) analysis and Kyoto Encyclopedia of Genes and Genomes (KEGG) enrichment analysis for differentially expressed genes were both implemented by KOBAS software (version 2.1.1).

The RNA-seq data of this study are publicly available in the NCBI Gene Expression Omnibus (GEO) under accession number: GSE216546. The related data are provided in Tables S4 and S5 in Supplementary Materials.

### 2.12. Statistical Analyses

All statistical analyses were performed using GraphPad Prism version 8.0. Data are presented as the means ± SD from at least three independent experiments. Statistical significance and the number of samples are indicated in the figures. A two-tailed Student’s *t*-test was used to compare differences among groups unless otherwise specified in the figure legends. For all tests, *p* < 0.05 was considered statistically significant.

## 3. Results

### 3.1. Que Prevents Hyperproliferation of ISCs in Aged Drosophila 

The ISCs in old *Drosophila* exhibit abnormal proliferation and differentiation, resulting in a progressive increase in the number of ISCs and progenitor cells [38]. To investigate whether Que can prevent age-related ISCs dysfunction in *Drosophila* midguts, we fed middle-aged (26-day-old) flies with a diet supplemented with Que for 14 days (Figure 1B). The ISCs and progenitor cells of the posterior midguts were then examined using a reporter line (*esg-GFP/CyO*), in which green fluorescent protein (GFP) is expressed under the control of the *escargot* (*esg*) gene. The results indicated a significant decrease in the number of *esg*^+^ cells in the aged flies (40-day-old) with Que supplementation compared to the flies without Que supplementation (Figure 1C), and this was not caused by the apoptosis of *esg-GFP*^+^ cells (Figure S1A–C). Among the four tested concentrations (1, 10, 50, and 100 µM), 10 µM Que showed the optimal effect (Figure 1C). In addition, we counted the number of Dl^+^ (ISC marker) cells and pH3^+^ (Phosphorylated-histone 3, a specific marker used to detect the proliferating cells) cells. It was found that aged flies supplemented with 10 µM Que had fewer Dl^+^ (Figure 1D–H) and pH3^+^ (Figure 1I) cells than control flies. We also found that Que supplementation can increase the number of ECs in aged flies, but there was no significant difference in the number of EEs (Figure S1D,E). These results suggest that Que supplementation can prevent the hyperproliferation of ISCs and gut hyperplasia in aged *Drosophila*.

### 3.2. Que Promotes ISCs Recovery and Improves Stress Tolerance in Drosophila

**Figure 2:**
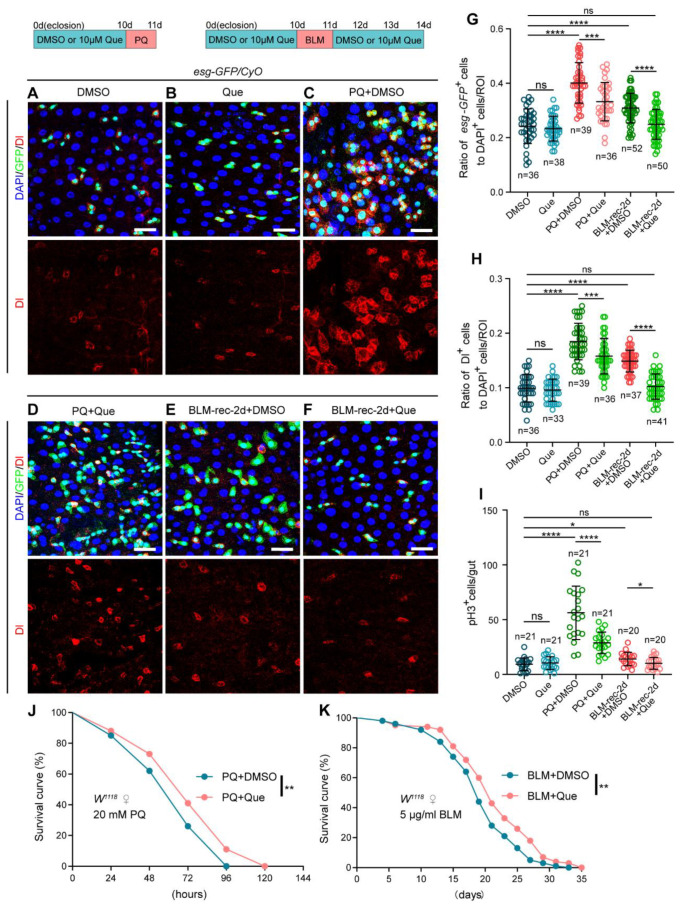
Que prevents stimulus-induced hyperproliferation of ISCs and improves stress tolerance in *Drosophila*. (**A**–**F**) Representative immunofluorescence images of *Drosophila* posterior midguts stained with DAPI, GFP, and Dl for DMSO (**A**), Que (**B**), PQ + DMSO (**C**), PQ + Que (**D**), BLM-rec-2d + DMSO (**E**), and BLM-rec-2d + Que (**F**) groups. The 0-day-old flies were fed with 10 µM Que for 10 days, then treated with 10 mM PQ for 1 day, followed by dissection. The 0-day-old flies were fed with 10 µM Que for 10 days, then treated with 25 µg/mL BLM for 1 day, and then resumed feeding with 10 µm Que for 1/2/3 days, followed by dissection. The top panels represent the merged images, whereas the bottom panels represent Dl. Scale bars represent 25 µm. (**G**,**H**) The ratio of *esg-GFP*^+^ cells (**G**) and Dl^+^ cells (**H**) to DAPI^+^ cells per ROI in the posterior midguts of flies in experiments (**A**–**F**). n: number of ROI counted. (**I**) The number of pH3^+^ cells in the whole gut of flies in experiments (**A**–**F**). n: number of gut counted. (**J**,**K**) Survival percentage of female *W^1118^* flies with DMSO (blue curve) or Que (pink curve) supplementation under 20 mM PQ (J) or 5 µg/mL BLM (**K**) treatments. Each group had 100 flies and three independent experiments were conducted. Error bars represent SDs. Log-rank test was used for lifespan analysis. Student’s *t*-tests, * *p* < 0.05, ** *p* < 0.01, *** *p* < 0.001, **** *p* < 0.0001, and non-significance (ns) represents *p* > 0.05.

Organism aging stems from complex genetic and epigenetic programs, which are caused, in part, by stressful events [39]. Given that Que had a positive impact on the gut of aged *Drosophila*, we explored whether Que supplementation could enhance the resistance of the *Drosophila* intestinal epithelium to deleterious events. Paraquat (PQ), a compound that induces toxicity by stimulating ROS production, has been widely employed to induce oxidative stress [40]. Results showed that there was no significant difference between the control group and the Que feeding group in young flies, while PQ treatment caused serious intestinal damage and ISCs were over-activated (Figure 2A–C,G–I). Que supplementation significantly restrained the hyperproliferation of ISC induced by PQ (Figure 2D,G–I), which is consistent with the powerful antioxidant capacity of Que. In addition, we found that Que can increase the number of ECs but did not affect the number of EEs in PQ-treated flies (Figure S2A,B). In the *Drosophila* intestine, ISCs can activate within hours in response to environmental stimuli, thereby repairing the damaged epithelium in a rapid and efficient manner [26,27]. Next, we examined whether Que supplementation could affect the regeneration processes of the damaged *Drosophila* intestine. Bleomycin (BLM), a DNA-damaging agent, was employed to damage the intestinal epithelium [41]. A previous study reported that the number of *esg-GFP*^+^, Dl^+^, and pH3^+^ cells is significantly elevated after exposure to BLM for 24 h (BLM-1d), but the damaged *Drosophila* intestine can recover to normal within 72 h (BLM-rec-3d) [42]. Interestingly, the present study found that the number of *esg-GFP*^+^, Dl^+^, and pH3^+^ cells in *Drosophila* with Que supplementation decreased to normal levels after recovery for two days (BLM-rec-2d) (Figure 2A,B,E–I), suggesting that Que could promote the regeneration processes of the impaired *Drosophila* gut. Similarly, Que supplementation can increase the number of ECs but did not affect the number of EEs in BLM-rec-2d flies (Figure S2A,B). Moreover, it was found that Que significantly increased the tolerance of *Drosophila* to PQ treatment and improved the survival rate of flies continuously exposed to a low concentration of BLM (Figure 2J,K). In summary, these findings suggest that Que supplementation can effectively protect ISCs from harmful stimuli and increase the tolerance of *Drosophila* to stressful insults.

### 3.3. Que Prevents Age-Related Degeneration of Gut Function and Extends the Lifespan in Drosophila

**Figure 3:**
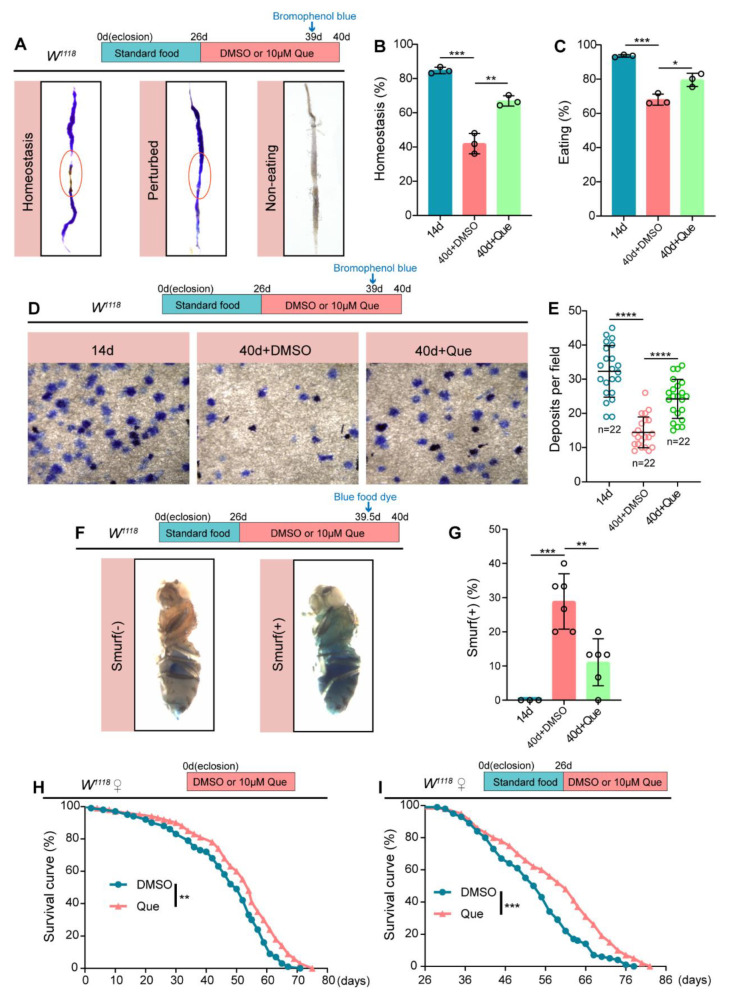
Que prevents age-related gut dysfunction and prolongs the lifespan. (**A**) Representative images of the intestinal acid-base homeostasis and the non-eating intestine of 14- and 40-day-old flies without Que supplementation and 40-day-old flies with 10 µM Que supplementation. A pink ellipse indicates the CCR. “Homeostasis” refers to CCR as yellow and “Perturbed” refers to CCR as blue. “Non-eating” means the flies did not eat, and the guts are not stained with bromophenol blue. (**B**,**C**) The percentage of acid-base balanced intestines (**B**) and eating intestines (**C**) in experiment (**A**). N ≥ 50 flies per group. Three independent experiments were conducted. (**D**,**E**) Representative images (**D**) and quantification (**E**) of excretion deposits of 14-and 40-day-old flies without Que supplementation and 40-day-old flies with 10 µM Que supplementation. Each group included 30 flies and three independent experiments were conducted. n: number of field counted. (**F**,**G**) Representative images (**F**) and quantification (**G**) of the percentage of the smurf flies in 14- and 40-day-old flies without Que supplementation and 40-day-old flies with 10 µM Que supplementation. Smurf (+) refers to a fly that leaks the blue dye from the gut into other tissues. Each group included 15 flies and three independent experiments were conducted. (**H**,**I**) Survival percentage of female *W^1118^* flies with DMSO (blue curve) or Que (pink curve) supplementation starting from eclosion (0-day-old) or middle age (26-day-old). Each group included 100 flies. Three independent experiments were conducted. Error bars represent SDs. Log-rank test was used for lifespan analysis. Student’s *t*-tests, * *p* < 0.05, ** *p* < 0.01, *** *p* < 0.001, and **** *p* < 0.0001.

The intestine is a vital organ with many physiological functions, including food ingestion and digestion, absorption of nutrients, excretion of waste products, and immune defense [43,44]. Age-related dysfunction of ISCs influences intestinal functions and impairs the intestinal barrier [45]. Considering that Que could prevent the age-related deterioration of ISCs, we further evaluated whether it could protect the intestinal functions of *Drosophila* upon aging. In *Drosophila* middle midguts, the copper cell region (CCR) secreting acid can be detected using bromophenol blue, the pH indicator. Notably, the size and function of CCR decline with age, disrupting intestinal acid-base homeostasis [35,46]. Herein, we found that Que supplementation could effectively protect the intestinal acid-base homeostasis in aged *Drosophila* (Figure 3A,B). In addition, the results showed that Que could improve food ingestion (Figure 3C) and excretion of waste products in aged *Drosophila* (Figure 3D,E). The integrity of the intestinal barrier contributes to maintaining epithelial homeostasis, defense against pathogens, and immune tolerance to commensal bacteria [47]. To investigate the effect of Que on the intestinal barrier, we performed a smurf assay where flies were fed on a diet mixed with non-absorbable blue dye; if the dye leaks from the intestine into other tissues, it indicates compromised intestinal barrier integrity [37]. Results showed that Que protected the integrity of the intestinal barrier in aged flies (Figure 3F,G). Since previous studies have revealed the critical role of intestinal regenerative capacity in extending lifespan [10], we also explored whether Que could extend the lifespan of *Drosophila*. The obtained results demonstrated that Que feeding prolongs the *Drosophila* lifespan whether fed from eclosion (0-day-old) or middle age (26-day-old) (Figure 3H,I). Altogether, these findings suggest that Que supplementation can prevent the age-related decline of intestinal function and prolong the lifespan of *Drosophila*.

### 3.4. Que Prevents Age-Associated Hyperproliferation of ISCs Partially through Its Antioxidant Activity

**Figure 4:**
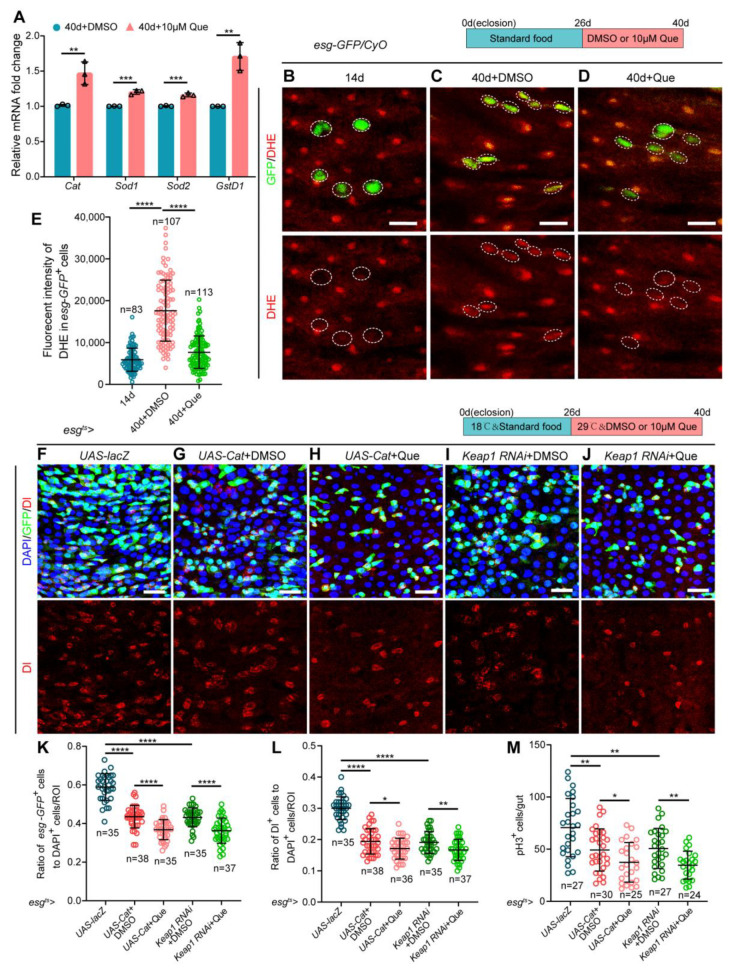
Que prevents age-related hyperproliferation of ISC partially through its antioxidative ability. (**A**) RT-qPCR analysis of the antioxidant-related genes (*Cat*, *Sod1*, *Sod2*, and *GstD1*) in the midguts of 40-day-old flies fed with or without Que. Three independent experiments were conducted. (**B**–**D**) Representative immunofluorescence images of the posterior midguts of 14- (**B**) and 40-day-old (**C**) flies without Que supplementation and 40-day-old flies with 10 µM Que supplementation (**D**) stained with DHE (red; ROS maker). *Esg-GFP*^+^ cells and DHE staining are circled by the white dashed line. The top panels represent the merged images, whereas the bottom panels represent DHE staining. Scale bars represent 10 µm. (**E**) Quantitation of DHE fluorescence intensity in *esg-GFP*^+^ cells from experiments (**B**–**D**). Each dot indicates one *esg-GFP*^+^ cell. (**F**–**J**) Representative immunofluorescence images of posterior midguts of flies carrying *esg^ts^-Gal4*-driven *UAS-lacZ* (**F**), *UAS-Cat* + DMSO (**G**), *UAS-Cat* + Que (**H**), *Keap1 RNAi* + DMSO (**I**), and *Keap1 RNAi* + Que (**J**) stained with DAPI, GFP, and Dl. The top panels represent the merged images, whereas the bottom panels represent Dl. Scale bars represent 25 µm. (**K**,**L**) The ratio of *esg-GFP*^+^ cells (**K**) and Dl^+^ cells (**L**) to DAPI^+^ cells per ROI in the posterior midguts of flies in experiments (**F**–**J**). n: number of ROI counted. (**M**) The number of pH3^+^ cells in the whole gut of flies in experiments (**F**–**J**). n: number of gut counted. Error bars represent SDs. Student’s *t*-tests, * *p* < 0.05, ** *p* < 0.01, *** *p* < 0.001, and **** *p* < 0.0001.

Accumulating evidence suggests that Que can prevent and treat various diseases, in which its antioxidant activity plays a significant role [48]. In addition, studies have demonstrated that elevated ROS increases the proliferation of ISCs, thereby leading to gut dysplasia in aged *Drosophila* [49,50]. Herein, real-time quantitative PCR (RT-qPCR) analyses were performed to explore whether Que promotes the homeostasis maintenance of ISCs via its antioxidant capacity. Results revealed that the expression of antioxidant-related genes (*Cat*, *Sod1*, *Sod2*, and *GstD1*) was elevated in the Que-fed aged flies, but not in young flies (Figure 4A and Figure S3A), indicating that Que has an aging-specific effect to scavenge ROS. Meanwhile, we directly characterized the ROS levels in *esg-GFP*^+^ cells using the fluorescent probe DHE, which exhibits increased fluorescence intensity upon oxidation [32]. Results showed that the intensity of DHE fluorescence was significantly elevated in the *esg-GFP*^+^ cells of aged flies, whereas aged flies subjected to Que feeding showed a significantly reduced DHE activity (Figure 4B–E). 

Moreover, we overexpressed *Cat* or depleted *Keap1* (a vital regulator of the intracellular redox state, the RNAi-mediated knockdown efficiency is shown in Figure S3B) under the control of *esg-Gal4* using the temperature-sensitive system (*esg^ts^-Gal4*) to prevent excessive ROS accumulation in aged guts [32]. The results indicated reduced numbers of *esg-GFP*^+^, Dl^+^, and pH3^+^ cells in these flies (Figure 4F,G,I,K–M). If Que maintained ISCs homeostasis exclusively through its antioxidant effect, it could not further enhance the gut phenotype produced by *Cat* overexpression or *Keap1* depletion. However, fewer *esg-GFP*^+^, Dl^+^, and pH3^+^ cells were detected after Que supplementation (Figure 4H,J,K–M). These results suggest that Que contributes to the homeostasis maintenance of ISCs in aged *Drosophila* partly through its antioxidant capacity.

### 3.5. Que Prevents Age-Associated Hyperproliferation of ISCs by Regulating Metabolic Pathways

**Figure 5:**
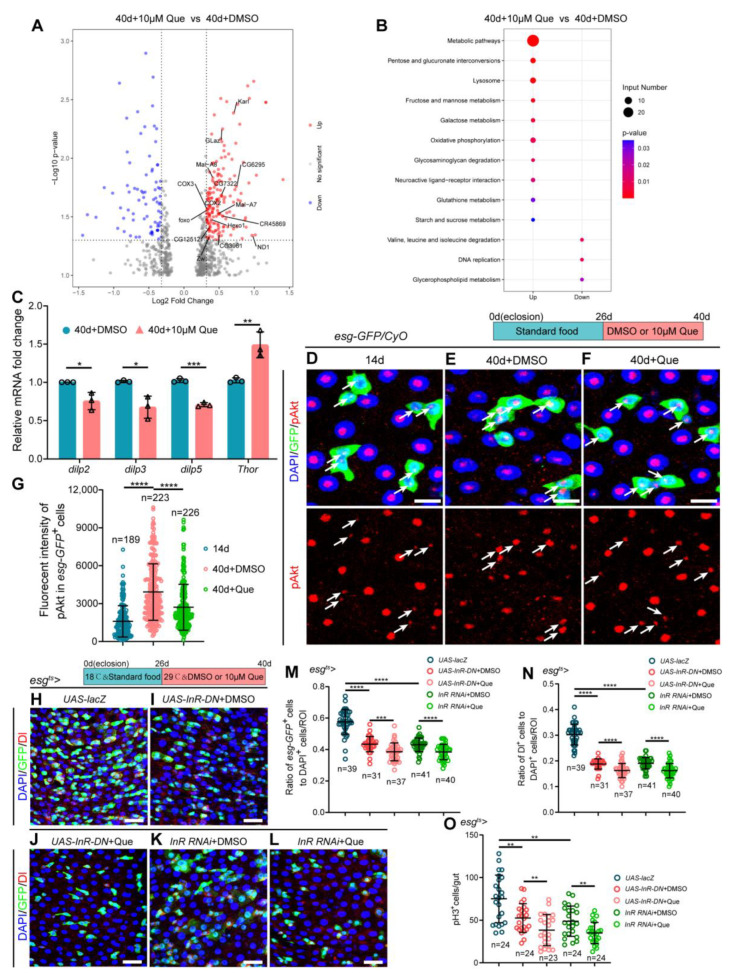
Que prevents age-related hyperproliferation of ISC by inhibiting insulin signaling. (**A**) Volcano plot shows differentially expressed genes in Que-treated 40-day-old flies compared to the control group. Red dots indicate significantly upregulated genes, blue dots indicate significantly downregulated genes, and gray dots indicate genes that are not significantly different. *Glaz*, *Karl*, and *foxo* are antioxidant-related genes, and the other identified genes are glucose and lipid metabolism-related. (**B**) KEGG pathway enrichment analysis of upregulated or downregulated genes in pair-wise comparison of 40-day-old flies without Que supplementation to 40-day-old flies with 10 µM Que supplementation. Both the p-value and input number represent the significance of the respective pathway. (**C**) RT-qPCR analysis of insulin signaling pathway-related genes (*dilp2*, *dilp3*, *dilp5*, and *Thor*) in the *esg-GFP*^+^ cells of 40-day-old flies fed with or without Que. Three independent experiments were conducted. (**D**–**F**) Representative immunofluorescence images of the posterior midguts of 14- (**D**) and 40-day-old (**E**) flies without Que supplementation and 40-day-old flies with 10 µM Que supplementation (**F**) stained with DAPI, GFP, and pAkt (red; a marker indicates activated insulin signaling pathway). White arrows indicate *esg-GFP*^+^ cells and pAkt staining. The top panels represent the merged images, whereas the bottom panels represent pAkt. Scale bars represent 10 µm. (**G**) Quantitation of pAkt fluorescence intensity in *esg-GFP*^+^ cells from experiments (**D**–**F**). Each dot indicates one *esg-GFP*^+^ cell. (**H**–**L**) Representative immunofluorescence images of the posterior midguts of flies carrying *esg^ts^-Gal4*-driven *UAS-lacZ* (**H**), *UAS-InR-DN* + DMSO (**I**), *UAS-InR-DN* + Que (**J**), *InR RNAi* + DMSO (**K**), and *InR RNAi* + Que (**L**) stained with DAPI, GFP, and Dl. Scale bars represent 25 µm. (**M**,**N**) The ratio of *esg-GFP*^+^ cells and Dl^+^ cells to DAPI^+^ cells per ROI in the posterior midguts of flies in experiments. (**H**–**L**). n: number of ROI counted. (**O**) The number of pH3^+^ cells in the whole gut of flies in experiments (**H**–**L**). n: number of gut counted. Error bars represent SDs. Student’s *t*-tests, * *p* < 0.05, ** *p* < 0.01, *** *p* < 0.001, and **** *p* < 0.0001.

To further explore the underlying mechanism through which Que maintains ISC homeostasis in aged flies, we performed RNA-sequencing of the midguts of old flies fed with and without Que. Results showed that antioxidant-related genes (*Glaz*, *Karl*, and *foxo*) were significantly upregulated in the midguts of aged *Drosophila* fed on a diet supplemented with Que (Figure 5A), which was consistent with the above results (Figure 4A). In addition, it was found that some genes associated with glucose and lipid metabolism were upregulated in aged *Drosophila* fed with Que (Figure 5A). Moreover, the KEGG enrichment analysis of differentially expressed genes revealed that the genes involved in metabolic pathways were significantly enriched (Figure 5B). Considering that the insulin/insulin-like growth factor signaling (IIS) pathway is the primary pathway that regulates energy metabolism, and previous studies have demonstrated that the IIS pathway plays an essential role in lifespan regulation [51,52], we speculated that Que might maintain the homeostasis of ISCs through regulating the insulin signaling pathway. To test this hypothesis, RT-qPCR was performed using sorted *esg^+^* cells to determine the expression of insulin signaling pathway-related genes in ISCs. Results revealed decreased expression of *dilp2*, *dilp3*, and *dilp5* (*Drosophila* insulin-like peptide 2, 3, and 5, which negatively regulate lifespan [53]), and increased expression of *Thor* (also known as *4EBP*) in aged flies fed with Que (Figure 5C), but not in young flies (Figure S4A), suggesting that Que regulates the insulin signaling pathway. Besides, we directly measured the activity of the insulin signaling pathway by evaluating the expression of phosphorylated Akt (p Akt). It was found that the fluorescence intensity of pAkt was significantly elevated in aged *esg-GFP*^+^ cells (Figure 5D,E,G). However, old flies subjected to Que supplementation showed a significantly reduced pAkt expression (Figure 5F,G), which indicated attenuated insulin signaling activity. To further verify the effect of Que on the insulin signaling pathway, we inhibited the pathway by expressing a dominant negative (DN) form of *InR* (insulin-like receptor) or expressing dsRNA for RNAi of *InR* (the RNAi-mediated knockdown efficiency is shown in Figure S4B), in which the numbers of *esg-GFP*^+^, Dl^+^, and pH3^+^ cells were fewer than in the control group (Figure 5H,I,K,M–O). Results revealed that Que supplementation could further reduce the number of *esg-GFP*^+^, Dl^+^, and pH3^+^ cells in aged flies (Figure 5J,L,M–O), probably due to its additional antioxidant capacity. Altogether, these findings suggest that Que contributes to the homeostasis maintenance of ISCs in aged *Drosophila*, in part, by inhibiting the insulin signaling pathway.

### 3.6. Que Prevents ISCs Aging via Inhibiting Insulin Signaling Pathway and Scavenging ROS

**Figure 6:**
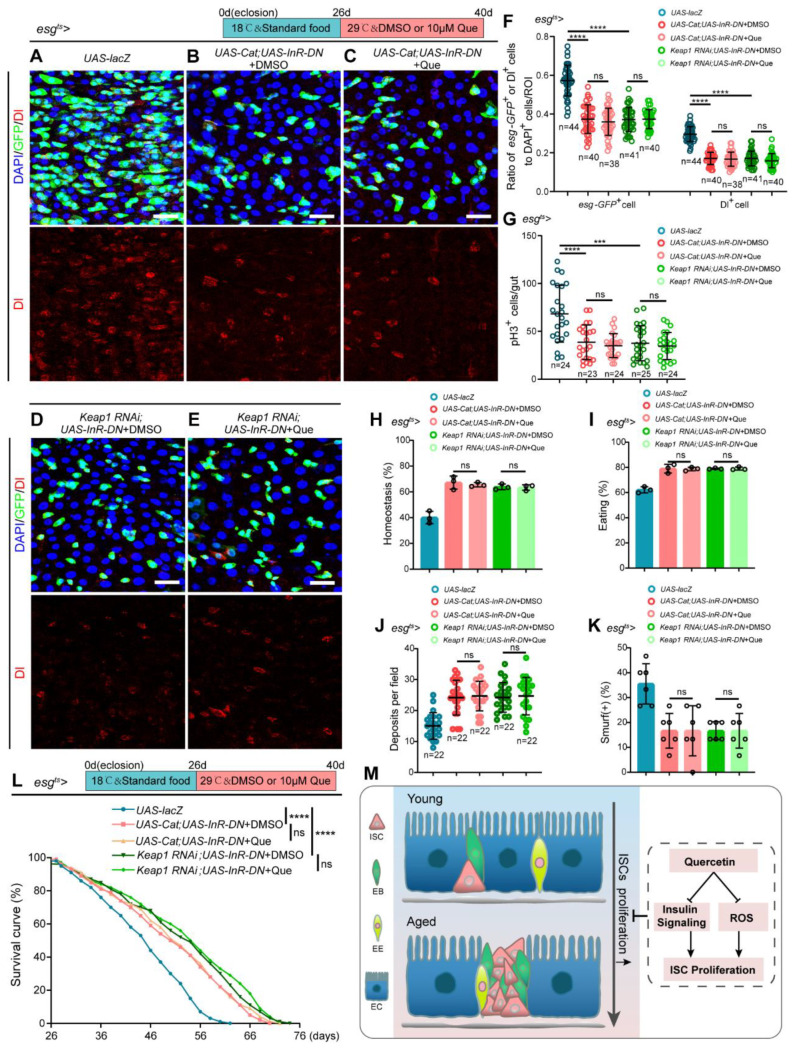
Que prevents age-related hyperproliferation of ISC through scavenging ROS and inhibiting insulin signaling pathway. (**A**–**E**) Representative immunofluorescence images of posterior midguts of flies carrying *esg^ts^-Gal4*-driven *UAS-lacZ* (**A**), *UAS-Cat; UAS-InR-DN* + DMSO (**B**), *UAS-Cat; UAS-InR-DN* + Que (**C**), *Keap1 RNAi; UAS-InR-DN* + DMSO (**D**), and *Keap1 RNAi; UAS-InR-DN* + Que (**E**) stained with DAPI, GFP, and Dl. The top panels represent the merged images, whereas the bottom panels represent Dl. Scale bars represent 25 µm. (**F**) The ratio of *esg-GFP*^+^ cells and Dl^+^ cells to DAPI^+^ cells per ROI in the posterior midguts of flies in experiments (**A**–**E**). n: number of ROI counted. (**G**) The number of pH3^+^ cells in the whole gut of flies in experiments (**A**–**E**). n: number of gut counted. (**H**,**I**) The percentage of acid-base balanced intestines (**H**) and eating intestines (**I**) of flies in experiments (**A**–**E**). N ≥ 50 flies per group. Three independent experiments were conducted. (**J**) Quantifying the excretion deposits of flies in experiments (**A**–**E**). Each group included 30 flies and three independent experiments were conducted. n: number of field counted. (**K**) The percentage of the smurf (+) flies in experiments (**A**–**E**). Each group included 15 flies and three independent experiments were conducted. (**L**) Survival percentage of indicated genotypes of female flies. Each group included 100 flies. Three independent experiments were conducted. (**M**) Schematic model of the mechanism of quercetin action. Que prevents the hyperproliferation of ISCs via attenuating oxidative stress and inhibiting the insulin signaling pathway. Error bars represent SDs. Log-rank test was used for lifespan analysis. Student’s *t*-tests, *** *p* < 0.001, **** *p* < 0.0001, and non-significance (ns) represents *p* > 0.05.

To evaluate whether Que prevents the age-related functional decline of ISCs by scavenging ROS and inhibiting insulin signaling pathway, we constructed two *Drosophila* strains: *esg^ts^*-*Gal4*-driven *Cat* overexpression and *InR-DN* overexpression and *esg^ts^*-*Gal4*-driven *Keap1* depletion and *InR-DN* overexpression. Results revealed a significant reduction of *esg-GFP*^+^, Dl^+^, and pH3^+^ cells in these flies compared to the normal aged flies (Figure 6A,B,D,F,G), and Que supplementation could not further enhance these effects (Figure 6C,E–G). In addition, there were no significant differences in intestinal acid-base homeostasis, the function of food ingestion and excretion of waste products, and the intestinal barrier between Que-fed and non-Que-fed aged flies (Figure 6H–K). Furthermore, Que supplementation could not further extend the lifespan (Figure 6L). These results suggest that Que prevents the age-associated functional decline of ISCs via scavenging ROS and inhibiting the insulin signaling pathway (Figure 6M).

## 4. Discussion

Over the past few decades, scientists in aging research have focused on understanding aging and identifying elaborate treatment strategies to delay aging and alleviate aging-related diseases. This study has shown that quercetin, a natural polyphenolic flavonoid, exerts excellent effects in maintaining intestinal homeostasis and prolonging the lifespan of aged *Drosophila*. Previous studies have demonstrated that Que is effective against several aging-associated disorders, including cancer [54], metabolic diseases [55], cardiovascular diseases [56], and Alzheimer’s disease (AD) [57]. However, the mechanisms underlying the beneficial effects of Que on aging-related diseases have not yet been elucidated. Herein, we found that Que prevents the age-associated functional decline of ISCs in aged *Drosophila* via scavenging ROS and inhibiting the insulin signaling pathway (Figure 6M).

Aging is a process characterized by the gradual deterioration of organism functions, accompanied by an increased risk of various diseases. In *Drosophila*, the ISCs exhibit over-proliferation and mis-differentiation during aging, thereby disrupting intestinal homeostasis and impairing intestinal function [7]. Results obtained in the present study indicated that Que could restrain ISCs dysfunction, prevent gut hyperplasia, maintain intestinal acid-base homeostasis and intestinal barrier integrity, and improve food intake and excretion functions in aged *Drosophila*. In addition, it was found that Que supplementation was effective against PQ-induced intestinal oxidative damage, could accelerate the recovery of damaged ISCs caused by BLM, and improve the survival rate of *Drosophila* under stressful stimuli. However, given that PQ and BLM can induce systemic damage and Que also exerts beneficial effects outside the gut, the increased survival of flies under these stressful stimuli may also be attributed to the effects of Que on other tissues. Moreover, the mechanism through which Que maintains ISC function under stressful stimuli may differ from that of natural aging, and thus there is a need for further investigation.

ROS, a class of reactive molecules derived from molecular oxygen, is often considered a double-edged sword [58]. On the one hand, appropriate ROS level functions as signaling molecules to regulate a series of critical physiological processes in organisms [59,60]. On the other hand, excessive ROS can lead to severe oxidative stress, ultimately causing organismal aging and various aging-related diseases [61,62,63]. In addition, mounting evidence has shown the important role of ROS in regulating stem cell homeostasis [64,65], whereas elevated ROS has been shown to increase the proliferation of *Drosophila* intestinal stem cells, thereby leading to intestinal damage and dysplasia [49,50]. This study found that Que contributes to the maintenance of intestinal homeostasis in aged *Drosophila*, at least in part, through its antioxidant activity. However, further studies should be conducted to explore the specific mechanism through which Que acts on the ROS signaling pathway.

The insulin/insulin-like growth factor (IGF) signaling (IIS), a nutrient-sensing pathway highly conserved in *Drosophila* and mammals, regulates various physiological processes, including growth, development, metabolism, reproduction, stress response, and aging [52,66,67]. Numerous studies have reported that reduced IIS prolongs the lifespan in diverse species, including worms, *Drosophila*, mouse, and human, whereas increased IIS shortens lifespan and increases the risk of age-associated diseases [51,52]. In addition, the IIS pathway plays a significant role in maintaining stem cell homeostasis [68,69,70]. *Drosophila* insulin-like peptides (DILPs), some evolutionarily conserved proteins that signal through insulin receptors, have been reported to regulate germline stem cell proliferation [30]. A previous study also showed that insulin is vital in the maintenance of neural stem cells in zebrafish [71]. Moreover, it has been reported that IIS regulates the proliferation and differentiation of diverse human stem cells, including mesenchymal stem cells, neural stem cells, and embryonic stem cells [72,73]. This study has demonstrated that Que contributes to the maintenance of intestinal stem cell (ISC) function in aged *Drosophila* partly through inhibiting the insulin signaling pathway. We found that the expression of some insulin signaling-related genes was altered in flow sorted *esg-GFP^+^* cells (Figure 5C), but not in the RNA seq data, possibly because the information of ISCs was covered by other differentiated epithelial cells (ECs and EEs) in *Drosophila* midguts. There is a need for further studies to elucidate the specific mechanism through which Que regulates the pathway.

## 5. Conclusions

In summary, we demonstrated that Que supplementation could prevent ISCs aging, maintain intestinal homeostasis, and extend the lifespan of aged *Drosophila*. In addition, we found that Que could accelerate the recovery of the damaged intestine and improve the tolerance of *Drosophila* to stressful stimuli. Furthermore, results suggested that Que prevents the age-related functional decline of ISCs via scavenging ROS and inhibiting insulin signaling. In conclusion, our findings suggest that Que plays a significant role in delaying adult stem cell aging. 

## Data Availability

All of the data is contained within the article and the Supplementary Materials.

## Supplementary Materials

The following supporting information can be downloaded at: https://www.mdpi.com/article/10.3390/antiox12010059/s1, Figure S1: Que supplementation did not cause ISCs apoptosis. Figure S2: The effect of Que on the number of ECs or EEs in stress stimulated flies. Figure S3: RT-qPCR analysis of the antioxidant-related genes in the midgut of 14-day-old flies. Figure S4: RT-qPCR analysis of the insulin signaling-related genes in the midgut of 14-day-old flies. Table S1: The list of the *Drosophila* genotypes; Table S2: The list of the primary antibodies; Table S3: The list of the primers. Table S4: The list of gene accession numbers for all the genes used in Figure 5. Table S5: The list of the top 30 genes upregulated and the top 30 genes downregulated in Que-treated 40-day-old flies compared to the control group.
