# Supplementary material for: Quercetin Prevents Intestinal Stem Cell Aging via Scavenging ROS and Inhibiting Insulin Signaling in Drosophila
Source: Antioxidants (Basel). 2022 Dec 27;12(1):59. doi: 10.3390/antiox12010059 (PMC9854609; doi:10.3390/antiox12010059)
Supplement: Supplementary file 1 [file antioxidants-12-00059-s001.zip › Supplementary Files/Supplementary Materials.pdf]

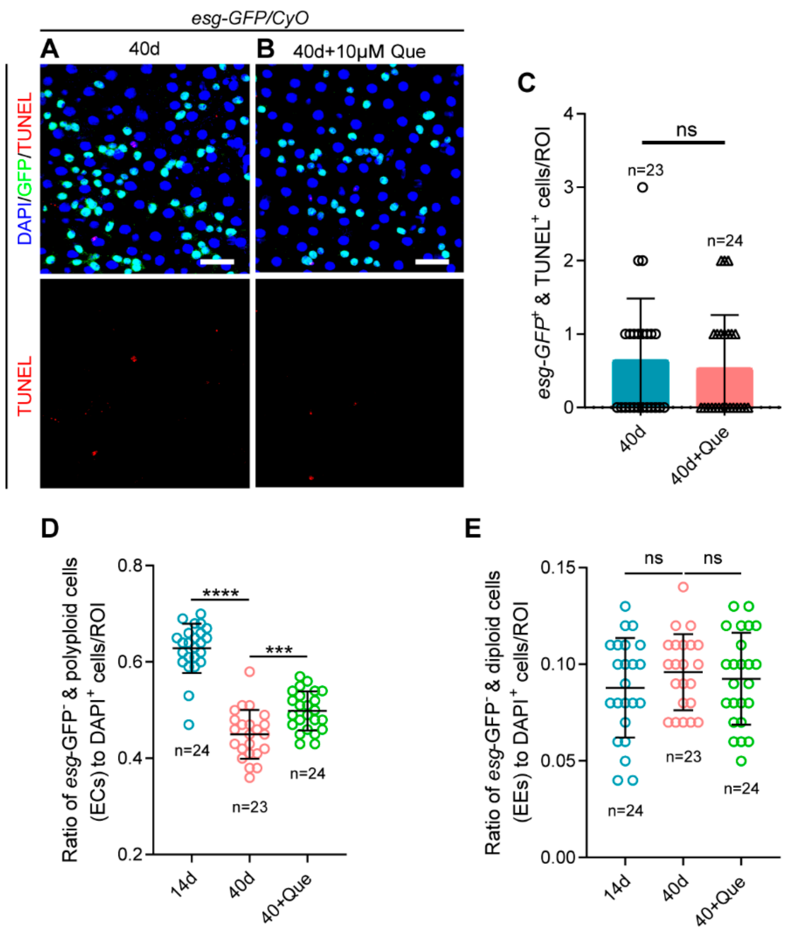

Figure S1: Que supplementation did not cause ISCs apoptosis. (A, B) Representative immunofluorescence images of 40-day-old (A) posterior midguts without Que supplementation and 40-day-old posterior midguts with 10 µM Que supplementation (B) stained with DAPI (blue; nuclei), GFP (green; ISCs and progenitor cells marker), and TUNEL (red; apoptosis marker). The top panels represent the merged images and the bottom panels represent TUNEL. Scale bars represent 25 µm. **(C)** The number of *esg-GFP*$^{+}$ & TUNEL^{+} cells per ROI in the posterior midguts of flies in experiments A, B. n: number of ROI counted. **(D, E)** The ratio of *esg*-GFP negative & polyploid cells (ECs) and *esg*-GFP negative & diploid cells (EEs) to DAPI^{+} cells per ROI in the posterior midguts of 14-day-old, 40-day-old flies without Que supplementation and 40-day-old flies with 10 µM Que supplementation. n: number of ROI counted.

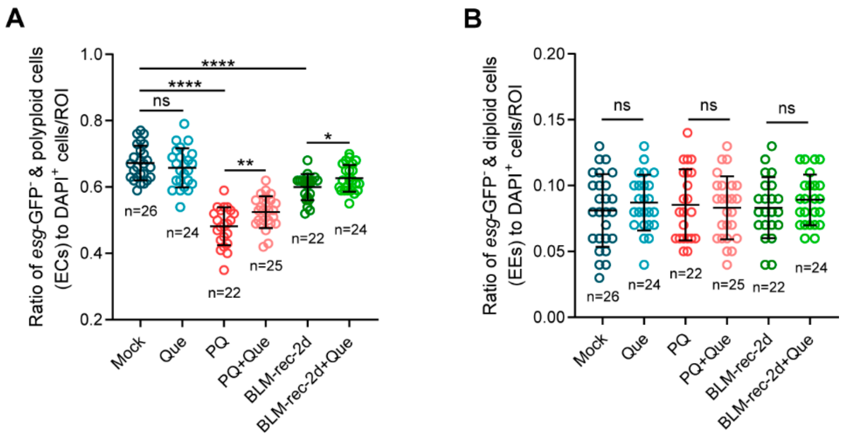

Figure S2: The effect of Que on the number of ECs or EEs in stress stimulated flies. (A, B) The ratio of *esg*-GFP negative & polyploid cells (ECs) and *esg*-GFP negative & diploid cells (EEs) to DAPI$^+$ cells per ROI in the posterior midguts of Mock (DMSO), Que, PQ + DMSO, PQ + Que, BLM-rec-2d + DMSO, and BLM-rec-2d + Que flies related to Figure 2A-F. n: number of ROI counted.

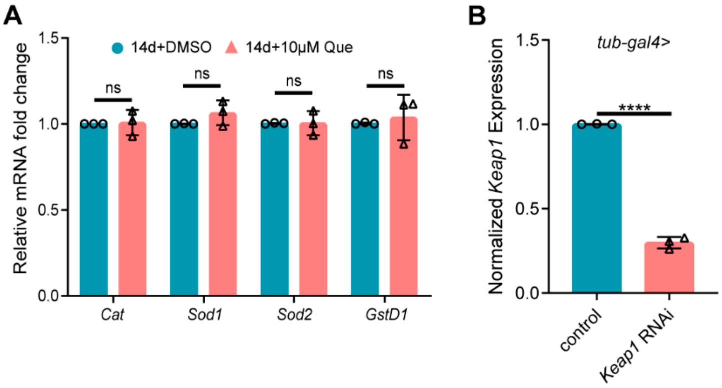

Figure S3: RT-qPCR analysis of the antioxidant-related genes in the midgut of 14-day-old flies. (A) RT-qPCR analysis of the antioxidant-related genes (*Cat*, *Sod1*, *Sod2*, and *GstD1*) in the midguts of 14-day-old flies fed with or without Que. Three independent experiments were conducted. **(B)** RT-qPCR analysis to show the RNAi-mediated knockdown efficiency for *Keap1 RNAi* line. Three independent experiments were conducted.

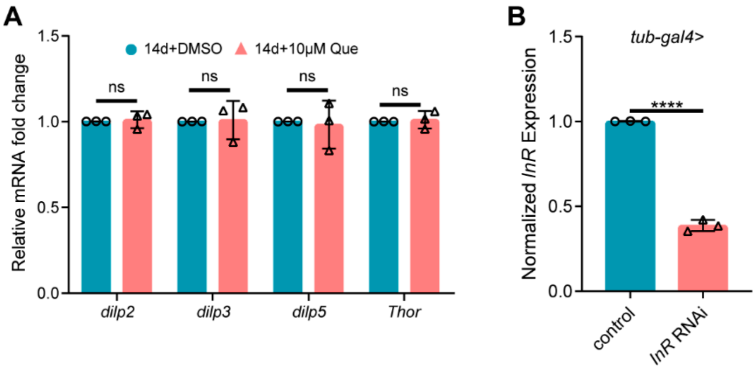

Figure S4: RT-qPCR analysis of the insulin signaling-related genes in the midgut of 14-day-old flies. (A) RT-qPCR analysis of insulin signaling pathway-related genes (*dilp2*, *dilp3*, *dilp5*, and *Thor*) in the *esg-GFP*$^{+}$ cells of 14-day-old flies fed with or without Que. Three independent experiments were conducted. **(B)** RT-qPCR analysis to show the RNAi-mediated knockdown efficiency for *InR RNAi* line. Three independent experiments were conducted.

**Table S1. The list of the *Drosophila* genotypes**	
**Figure 1**	
**D-G**	*w$^-$; esg-GFP/CyO; +/+*
**Figure 2**	
**A-F**	*w$^-$; esg-GFP/CyO; +/+*
**J, K**	*w$^-$; + /+; +/+*
**Figure 3**	
**A, D, F, H, I**	*w$^-$; + /+; +/+*
**Figure 4**	
**A**	*w$^-$; + /+; +/+*
**B-D**	*w$^-$; esg-GFP/CyO; +/+*
**F**	*w$^-$; esg-Gal4, UAS-GFP, tub-Gal80^{ts}/ UAS-lacZ; +/+*
**G, H**	*w$^-$; esg-Gal4, UAS-GFP, tub-Gal80^{ts}/UAS-Cat; +/+*
**I, J**	*w$^-$; esg-Gal4, UAS-GFP, tub-Gal80^{ts}/UAS-Keap1 RNAi; +/+*
**Figure 5**	
**A, B**	*w$^-$; + /+; +/+*
**C -F**	*w$^-$; esg-GFP/CyO; +/+*
**H**	*w$^-$; esg-Gal4, UAS-GFP, tub-Gal80^{ts}/ UAS-lacZ; +/+*
**I, J**	*w$^-$; esg-Gal4, UAS-GFP, tub-Gal80^{ts}/ +; UAS-InR-DN/+*
**K, L**	*w$^-$; esg-Gal4, UAS-GFP, tub-Gal80^{ts}/ UAS-InR RNAi; +/+*
**Figure 6**	
**A**	*w$^-$; esg-Gal4, UAS-GFP, tub-Gal80^{ts}/ UAS-lacZ; +/+*
**B, C**	*w$^-$; esg-Gal4, UAS-GFP, tub-Gal80^{ts}/UAS-Cat; UAS-InR-DN /+*
**D, E**	*w$^-$; esg-Gal4, UAS-GFP, tub-Gal80^{ts}/ UAS-Keap1 RNAi; UAS-InR-DN /+*

Table S2 (Reagent Table). The list of the primary antibodies		
**Reagent**	**Source**	**Dilution**
Chicken polyclonal anti-GFP	Abcam Cat# ab13970 RRID:AB_300798	1: 1000
Mouse anti-Delta	DSHB Cat# C594.9B RRID:AB_528194	1: 50
Rabbit anti-Phospho-Akt (Ser473)	Cell Signaling Cat# 4060S RRID:AB_ 2315049	1: 200
Rabbit anti-phosphoHistone H3 (Ser10)	Millipore Cat# 06-570 RRID:AB_310177	1: 1000

Table S3. The list of the primers	
**Target**	**Sequence**
Cat	F: 5’-TTCCTGTGGGCAAAATGGTG-3’<br>R: 5’-ATCTTCACCTTGTACGGGCA-3’
Sod1	F: 5’- CTGCTCTGCTACGGTCACAC-3’<br>R: 5’- ACAGCTTTAACCACCATTTCG-3’
Sod2	F: 5’- ATAAGCATCTGCGGATCGGA-3’<br>R: 5’- ACGCCTTTGTGAAGCGTTATC-3’
GstD1	F: 5’- CATCGCGAGTTTCACAACAG-3’<br>R: 5’- GTTGAGCAGCTTCTTGTTCAG-3’
dilp2	F: 5’- GGCCAGCTCCACAGTGAAGT-3’<br>R: 5’- TCGCTGTCGGCACCGGGCAT-3’
dilp3	F: 5’- AAGCTCTGTGTGTATGGCTT-3’<br>R: 5’- AGCACAATATCTCAGCACCT-3’
dilp5	F: 5’- TGTTCGCCAAACGAGGCACCTTGG-3’<br>R: 5’- CACGATTTGCGGCAACAGGAGTCG-3’
4EBP	F: 5’- CACTCCTGGAGGCACCA-3’<br>R: 5’- GAGTTCCCCTCAGCAAGCAA-3’
Rp49	F: 5’- ATCGGTTACGGATCGAACAAGC -3’<br>R: 5’- GTAAACGCGGTTCTGCATGAGC -3’
